# Diabetes mellitus in HIV-infected patients: fasting glucose, A1c, or oral glucose tolerance test – which method to choose for the diagnosis?

**DOI:** 10.1186/s12879-018-3221-7

**Published:** 2018-07-06

**Authors:** Ana Rita Coelho, Flávia Andreia Moreira, Ana Cristina Santos, André Silva-Pinto, António Sarmento, Davide Carvalho, Paula Freitas

**Affiliations:** 1 Medical Student. Faculty of Medicine, University of Porto. Alameda Prof. Hernâni Monteiro, 4200-319 Porto, Portugal; 2 EPIUnit - Instituto de Saúde Pública, Universidade do Porto, Porto, Portugal; 3 Departamento de Ciências da Saúde Pública e Forenses e Educação Médica, Faculdade de Medicina, Universidade do Porto, Porto, Portugal; 4 Infectious Diseases Department, Centro Hospitalar São João, Porto, Portugal; 5 Renal, Urological and Infectious Diseases Department, Faculty of Medicine of University of Porto, Porto, Portugal; 6 Endocrinology, Diabetes and Metabolism Department, Centro Hospitalar São João, and Faculty of Medicine, i3S - Instituto de Investigação e Inovação em Saúde, University of Porto, Porto, Portugal

**Keywords:** HIV infection, Diabetes mellitus diagnosis, Fasting glucose, Oral glucose tolerance test, HbA1c

## Abstract

**Background:**

Antiretroviral therapy dramatically reduced HIV-related morbidity and mortality, prolonging the lifespan of HIV-infected patients. Greater duration of infection and exposure to antiretroviral therapy makes these patients susceptible to traditional cardio-metabolic risk factors and pathologies. The optimal diagnostic protocol for Diabetes Mellitus in these patients is still controversial. Haemoglobin A1c (HbA1c) has been shown to underestimate glycaemia levels and the oral glucose tolerance test (OGTT) has been shown to reveal cases of glucose metabolism disturbances in patients with normal fasting glucose. Thus, this study aimed to determine the prevalence of prediabetes and diabetes in a population of HIV-infected patients undergoing combined antiretroviral therapy, using three different diagnostic methods (fasting glucose, OGTT and HbA1c), to determine the agreement between the different methods and the characteristics associated with each one.

**Methods:**

This study analyzed 220 HIV-infected patients on antiretroviral therapy. Patient characteristics were collected using a standardized protocol. Disturbances of glucose homeostasis were defined by the ADA 2017 criteria. Patients were characterized according to the presence or absence of clinical lipodystrophy, and distributed into four different categories, according to the presence, or absence of either clinical lipoatrophy, or abdominal prominence. Insulin resistance was assessed by HOMA-IR and QUICKI indexes. Agreement between the diagnostic methods was assessed by Cohen’s kappa coefficient.

**Results:**

There were no patients diagnosed with diabetes with HbA1c. 5.9% prevalence was obtained when OGTT was used, and 3.2% prevalence when fasting glucose was used. Prediabetes had a prevalence of 14.1% when using HbA1c, 24.1% when using OGTT, and 20% when using fasting glucose. In all three methods, glucose homeostasis disturbances were associated with older age and higher resistance to insulin. Regarding other characteristics, associations varied between the three methods. The agreement between them was fair, or slight.

**Conclusions:**

We observed that HbA1c was the method that diagnosed the least amount of cases and that OGTT was the one that diagnosed the most cases. Accordingly, our results indicate that HbA1c underestimated glycaemia levels in this population and that the use of OGTT might allow an earlier diagnosis of glucose homeostasis disturbances, potentially making it possible to avoid severe complications of DM.

## Background

Antiretroviral therapy (ART) dramatically reduces HIV-related morbidity and mortality and prolongs the lifespan of this infected population [1]. The greater survival of patients living with the infection makes them more susceptible to exposure to the same traditional cardio-metabolic risk factors and pathologies as the general population. These complications are likely not only correlated with age, but also with the cumulative exposure to ART [1–5].

Cardio-metabolic pathology is, in fact, becoming an increasing problem associated with HIV infection under ART [2, 5, 6]. In a large-scale HIV population study, De Wit et al. found that the incidence of new-onset diabetes mellitus (DM) increased with cumulative exposure to ART [5]. In fact, it has been shown that HIV populations can have up to a two-fold higher risk of DM when compared to the general population [4]. Besides ART, traditional risk factors have also been associated with the development of DM in this population, such as family history, obesity, older age, race, abdominal prominence (e.g. waist circumference) and statin use [4, 7, 8].

In the general population, DM has been identified as a high-risk, and very high-risk factor for the development of cardiovascular disease [9]. This was also demonstrated in the HIV population, where it was revealed that DM is a risk factor that substantially increases the chance of developing coronary heart disease, especially in cases of prolonged infection [6]. Thus, it is essential to regularly screen for this condition in this population, in order to prevent these kinds of complications.

The optimal diagnostic methodology for DM in HIV patients is still a controversial topic. The current European AIDS Clinical Society guidelines recommend the evaluation of fasting glucose as an initial assessment of glucose metabolism in patients with new HIV diagnostic and also prior to starting ART. An oral glucose tolerance test (OGTT), or haemoglobin A1c (HbA1c) measurement is only recommended if fasting glucose levels reveal prediabetes [10].

Accordingly, this study aims to determine the prevalence of prediabetes and DM using three differences diagnostic methods (fasting glucose, OGTT and HbA1c) in a population of HIV-infected patients under combined antiretroviral therapy. Additionally, it is our aim to investigate which characteristics differ in each diagnostic method between the different groups, and what is the agreement between the different methods.

## Methods

### Participants

As part of a cross-sectional study, between 2005 and 2016, 220 non-institutionalized HIV-infected adults, consecutively referred from the Infectious Diseases Department, were evaluated at the Endocrinology Outpatient Clinic of São João Hospital. Patients were included in the study on their first visit, and only patients on combined antiretroviral therapy were included. A history of previously-diagnosed DM and use of anti-diabetic therapy excluded patients from our study. The Ethics Committee for Health of Hospital São João approved this study and each patient provided written informed consent.

### Clinical assessment

For each patient the following information was collected using a standardized protocol: age, known duration of HIV infection and of combined antiretroviral therapy exposure, current type of ART, HIV infection risk factors and characterization of the infection, smoking history (past, current, or never), history of diabetes and hypertension, and use of anti-diabetic, anti-hypertensive, and lipid lowering drugs. We used the Centers for Disease Control and Prevention (CDC) criteria for classifying the degree of infection [11].

Weight, height and waist circumference were measured, and Body Mass Index (BMI) was also calculated. Body weight was measured using TANITA (Tanita®, model TBF 300), and scale and height was measured to the nearest centimeter in the standing position using a wall stadiometer (Holtain Limited Crymych, Dyfed®). BMI was calculated as weight divided by height squared (kg/m2). The waist circumference was measured midway between the lowest rib and iliac crest, at the end of a gentle expiration, with the patient standing upright, face directed forward and shoulders relaxed.

Clinical lipodystrophy was defined as peripheral lipoatrophy with or without central fat accumulation assessed by both patient and practitioner [12]. Patients with at least one light, moderate, or severe subjective lipoatrophic feature (identified by lipoatrophy-specific physical examination) were asked to report whether he/she had any change in fat in their cheeks, the side of their face, legs, arms, or buttocks. Patients were classified as being without peripheral lipoatrophy when none of the previously described features were present [13]. Presence of central fat accumulation or abdominal prominence was defined by the measurement of waist circumference using the International Diabetes Federation criteria for metabolic syndrome (waist circumference ≥ 94 cm for Europid men and ≥ 80 cm for Europid women). Patients were classified into four different categories, according to the presence or absence of either clinical lipoatrophy or abdominal prominence: 1) no lipodystrophy – patients without lipoatrophy and without abdominal prominence; 2) isolated central fat accumulation – patients without lipoatrophy and with abdominal prominence; 3) lipoatrophy – patients with lipoatrophy and without abdominal prominence; 4) mixed forms of lipodystrophy – patients with lipoatrophy and with abdominal prominence [13]. The clinical assessment was performed by the same practitioner (PF).

### Laboratory analysis

A venous blood sample was taken after a 12-h overnight fast. The 12-h overnight fast was confirmed with the participants prior to the collection of the blood sample. If the 12-h fasting period was not observed the blood sample collection was rescheduled. All the samples were analyzed at the central laboratory of our hospital. The measurements of total cholesterol, low-density lipoprotein cholesterol, high-density lipoprotein cholesterol, triglycerides, plasma glucose and HbA1c serum levels were determined using commercial kits. Hepatitis C was diagnosed by serological assays that detect antibody to hepatitis C virus (anti-HCV).

All patients without a previous diagnosis of diabetes were submitted to an OGTT. The OGTT was performed as described by the World Health Organization, using a glucose load containing the equivalent of 75 g anhydrous glucose dissolved in water.

The CD4 cell count was determined by flow cytometry and plasma HIV-1 RNA loads were measured by a quantitative reverse transcriptase polymerase chain reaction (Roche Diagnostic Systems, Inc., Branchburg, NJ, USA), which has a lower limit of detection of 50 copies/mL.

### Criteria for the definition of disturbances of glucose homeostasis

Disturbances of glucose homeostasis were defined by the American Diabetes Association 2017 criteria [14]. Patients were divided into three groups: no diabetes (No DM), prediabetes and diabetes (DM). No DM was defined as fasting glucose <100 mg/dL, HbA1c <5.7% or 120 min plasma glucose <140 mg/dL during the OGTT. Prediabetes was defined as fasting glucose between 100 and 126 mg/dL, HbA1c between 5.7 and 6.5% or 120 min plasma glucose between 140 and 200 mg/dL during the OGTT. DM was defined as fasting glucose ≥126 mg/dL, HbA1c ≥6.5% or 120 min plasma glucose ≥200 mg/dL during the OGTT.

### Measurements of insulin resistance

Insulin resistance was defined by the homeostasis model assessment of insulin resistance (HOMA), and insulin sensitivity by the quantitative insulin sensitivity check index (QUICKI). These indexes were calculated by the following formulas: HOMA-IR index = (fasting plasma insulin × fasting plasma glucose)/22.5 [15] and QUICKI = 1/[log (fasting insulin in mU/l) + log (fasting plasma glucose in mg/dL)] [16]. Glucose was expressed in mmol/L and insulin in μUI/mL. Insulin resistance was defined when the value of HOMA >4 [8].

### Statistical analysis

Quantitative variables were described as mean and standard deviation (SD), or median and interquartile range (IQR), and were compared using Student-t and ANOVA or Mann–Whitney and Kruskal-Wallis tests, as appropriate.

Categorical variables were described as counts and proportions, and compared using the chi-square or Fisher’s exact test. The kappa coefficient was computed to analyze statistical agreement between the three different diagnostic methods used for defining prediabetes and DM. Statistical analysis was performed using SPSS version 24.0 software (SPSS Inc., Chicago, Illinois, USA). All probabilities were two tailed, and *p* values of <0.05 were regarded as significant.

## Results

### Baseline characteristics

A total of 220 HIV-infected patients under ART were evaluated. The mean age of patients included was 45.8 ± 11.5 years, and 60.5% of them were males. All the demographic and clinical characteristics accessed in this study are presented in Table 1, according to the presence or absence of clinical lipodystrophy.

**Table 1:** Sample’s baseline characteristics, according to the presence of Clinical Lipodystrophy (CL)

	With CL	Without CL	*P* value
*n* (%)	115 (52.3)	105 (47.7)	
Sex [*n* (%)]			0.099
Male	76 (66.1)	57 (54.3)	
Female	39 (33.9)	48 (45.7)	
Age [years, mean (SD)]	47.5 (11.3)	43.8 (11.5)	0.017
Duration of HIV infection [years, median (IR)]	9 (5)	6 (6)	0.001
cART [years, median (IR)]	8 (5)	5 (5.5)	< 0.001
Weight [Kg, mean (SD)]	64.1 (12.8)	73.9 (12.6)	< 0.001
Height [m, mean (SD)]	1.7 (0.1)	1.7 (0.1)	0.765
BMI [(kg/m2), mean (SD)]	23.6 (3.8)	27.2 (4.5)	< 0.001
Waist circumference [cm, mean (SD)]	88.4 (10.8)	95.1 (12.1)	< 0.001
CD4 cell count [cells/mm3, median (IR)]	554 (385)	479 (300)	0.238
HIV RNA (<50) [*n* (%)]	100 (100)	92 (100)	
Hepatitis C co-infection [*n* (%)]	34 (29.8)	30 (29.4)	0.999
Hypertension [*n* (%)]	45 (39.1)	23 (21.9)	0.009
HIV risk factor [*n* (%)]			0.162
Intravenous drug user	1 (25)	3 (15.8)	
Homosexual contact	0 (0)	2 (10.5)	
Heterosexual contact	2 (50)	14 (73.7)	
Others	1 (25)	0 (0)	
CDC clinical categories [*n* (%)]			0.389
A	63 (54.8)	56 (53.3)	
B	1 (0.9)	4 (3.8)	
C	51 (44.3)	45 (42.9)	
ART [*n* (%)]			
IP	61 (53)	62 (59)	0.447
NNRTI	55 (47.8)	47 (44.8)	0.749
NRTI	113 (98.3)	97 (92.4)	0.051
Smoking history [*n* (%)]			0.032
Never	38 (33.3)	53 (50.5)	
Current	56 (49.1)	36 (34.3)	
Former	20 (17.5)	16 (15.2)	
Total cholesterol [mg/dL, mean (SD)]	221.9 (53.2)	227.8 (57.8)	0.433
LDL- cholesterol [mg/dL, mean (SD)]	129.4 (48.1)	140 (45.4)	0.097
HDL- cholesterol [mg/dL, mean (SD)]	46.5 (14.8)	49.5 (13.3)	0.123
Triglycerides [mg/dL, median (IR)]	215.5 (214.5)	171 (154)	0.007
Statin use [*n* (%)]	33 (28.7)	13 (12.4)	0.005
Fibrate use [*n* (%)]	41 (35.7)	24 (22.9)	0.054

*CL* clinical lipodystrophy, *cART* combination antiretroviral therapy, *BMI* body mass index, *ART* antiretroviral therapy, *PI* protease inhibitor, *NNRTI* non-nucleoside reverse transcriptase inhibitor, *NRTI* nucleoside reverse transcriptase inhibitor, *HOMA* homeostatic model assessment, *QUICKI* quantitative insulin sensitivity check index, *SD* standard deviation, *IR* interquartile range

Patients with clinical lipodystrophy were older [47.5 (11.29) vs 43.82 (11.49) years; *P* = 0.017], had longer duration of the HIV infection [9.0 (5.0) vs 6.0 (6.0) years; *p* = 0.001] and of ART use [8.0 (5.0) vs 5.0 (5.5) years; *p* < 0.001]. Regarding anthropometric measures, patients with clinical lipodystrophy had lower weight (*p* < 0.001), BMI ((*p* < 0.001), and waist circumference mean values (*p* < 0.001). Hypertension was more frequent in patients with clinical lipodystrophy [45 (39.1) vs 23 (21.9) %; *p* = 0.009], as were current smokers [56 (49.1) vs 36 (34.3) %; *p* = 0.032], and triglycerides median values were significantly higher than those of patients without clinical lipodystrophy [215.5 (214.5) vs 171 (154) mg/dL; *p* = 0.007]. With regards to pharmaceutical therapy, the use of statins (*p* = 0.005) and fibrates (*p* = 0.054) was more frequent in patients with clinical lipodystrophy.

No differences were found between patients with or without clinical lipodystrophy in terms of gender, CD4+ cell count, percentage of viral suppression, prevalence of co-infection with Hepatitis C, type of risk factor for the HIV transmission, CDC clinical categories, type of ART used, and lipid profile.

### Hemoglobin A1c

No patients were diagnosed with DM using this method (HbA1c ≥ 6.5%). Therefore, in Table 2, the results regarding two groups: no DM (HbA1c ≤ 5.7%) and prediabetes (5.7 < HbA1c < 6.5) are presented.

**Table 2:** Sample’s characteristics, according to the presence of no DM or prediabetes, accessed by HbA1c

	HbA1c		*P* value
	<5.7%	5.7–6.4%	
*n* (%)	189 (85.9)	31 (14.1)	
Sex [*n* (%)]			0.623
Male	116 (61.4)	17 (54.8)	
Female	73 (38.6)	14 (45.2)	
Age [years, mean (SD)]	45 (11.3)	50.4 (11.9)	0.016
Duration of HIV infection [years, median (IR)]	8 (6)	8 (7.0)	0.698
cART [years, median (IR)]	6 (6.5)	7 (7.0)	0.136
Clinical lipodystrophy [*n* (%)]			0.909
Without CL	91 (48.1)	14 (45.2)	
With CL	98 (51.9)	17 (54.8)	
Body Composition [*n* (%)]			0.469
No lipodystrophy	28 (15.3)	3 (10)	
Isolated central fat accumulation	59 (32.2)	10 (33.3)	
Lipoatrophy	52 (28.4)	6 (20)	
Mixed form of lipodystrophy	44 (24)	11 (36.7)	
BMI [(kg/m2), mean (SD)]	25 (4.5)	26.9 (4.5)	0.031
Waist circumference [cm, mean (SD)]	90.9 (11.6)	95.2 (13.2)	0.065
CD4 cell count [cells/mm3, median (IR)]	500 (345)	528 (312)	0.819
HIV RNA (<50) [*n* (%)]	165 (100)	27 (100)	
Hepatitis C co-infection [*n* (%)]	57 (30.8)	7 (22.6)	0.474
CDC clinical categories [*n* (%)]			0.093
A	107 (56.6)	12 (38.7)	
B	5 (2.6)	0 (0)	
C	77 (40.7)	19 (61.3)	
ART [*n* (%)]			
IP	106 (56.1)	17 (54.8)	0.999
NNRTI	88 (46.6)	14 (45.2)	0.999
NRTI	182 (96.3)	28 (90.3)	0.152
HOMA-IR index [median (IR)]	1.6 (1.5)	2.5 (5.1)	0.023
QUICKI index [median (IR)]	0.4 (0.1)	0.3 (0.1)	0.023
Total cholesterol [mg/dL, mean (SD)]	225.6 (55.4)	219 (55.8)	0.544
LDL- cholesterol [mg/dL, mean (SD)]	133.5 (47.5)	140.2 (44.6)	0.473
HDL- cholesterol [mg/dL, mean (SD)]	48.3 (14.4)	45.8 (11.8)	0.374
Triglycerides [mg/dL, median (IR)]	203 (198)	139 (133.8)	0.010
Statin use [*n* (%)]	41 (21.7)	5 (16.1)	0.640
Fibrate use [*n* (%)]	61 (32.3)	4 (12.9)	0.048

*DM* diabetes mellitus, *HbA1c* glycated haemoglobin, *CL* clinical lipodystrophy, *cART* combination antiretroviral therapy, *BMI* body mass index, *ART* antiretroviral therapy, *PI* protease inhibitor, *NNRTI* non-nucleoside reverse transcriptase inhibitor, *NRTI* nucleoside reverse transcriptase inhibitor, *HOMA* homeostatic model assessment index, *QUICKI* quantitative insulin sensitivity check index, *SD* standard deviation, *IR* interquartile range

In our population, 31 patients (14.1%) were diagnosed with prediabetes. These patients were older [50.35 (11.89) vs 44.99 (11.30) years; *p* = 0.016], and had a higher BMI [26.90 (4.46) vs 25.01 (4.49) kg/m2; *p* = 0.031], compared to the no DM patients, but had lower median level of triglycerides [139 (133.8) vs 203 (198.0) mg/dL, *p* = 0.010], and less frequently used fibrate [4(12.9) vs 61 (32.3)%; *p* = 0.048]. The values obtained for HOMA-IR were higher among patients with prediabetes, and the difference between the groups was statistically significant [2.51 (5.13) vs 1.62 (1.46); *p* = 0.023].

There were no differences between the diagnostic groups with regards to sex, duration of HIV infection or ART use, presence or absence of clinical lipodystrophy, body composition types, waist circumference, CD4 cell count, percentage of viral suppression, prevalence of hepatitis C coinfection, CDC clinical category, type of ART used, lipid profile, and frequency of use of statins.

### Oral glucose tolerance test

With regards to OGTT, 53 patients (24.1%) were diagnosed with prediabetes (140 mg/dL < glucose at 120 min < 200 mg/dL), and 13 patients (5.9%) with DM (glucose at 120 min ≥ 200 mg/dL). In Table 3, the results regarding the three categories of glucose homeostasis are presented.

**Table 3:** Sample’s characteristics according to the presence of no DM, prediabetes and DM, accessed by OGTT

	Glucose at 120 min			*P* value
	<140 mg/dL	140–200 mg/dL	≥200 mg/dL	
*n* (%)	154 (70)	53 (24.1)	13 (5.9)	
Sex [*n* (%)]				0.061
Male	101 (65.6)	26 (49.1)	6 (46.2)	
Female	53 (34.4)	27 (50.9)	7 (53.8)	
Age [years, mean (SD)]	43.4 (10.2)	50.4 (12.1)	55 (13.6)	< 0.001
Duration of HIV infection [years, median (IR)]	8 (6)	7 (6)	10 (8)	0.980
cART [years, [median (IR)]	6 (5.3)	6 (7)	9 (9)	0.566
Clinical lipodystrophy [*n* (%)]				0.148
Without CL	78 (50.6)	24 (45.3)	3 (23.1)	
With CL	76 (49.4)	29 (54.7)	10 (76.9)	
Body Composition [*n* (%)]				0.078
No lipodystrophy	23 (15.5)	7 (13.5)	1 (7.7)	
Isolated central fat accumulation	50 (33.8)	17 (32.7)	2 (15.4)	
Lipoatrophy	45 (30.4)	11 (21.2)	2 (15.4)	
Mixed form of lipodystrophy	30 (20.3)	17 (32.7)	8 (61.5)	
BMI [(kg/m2), [median (IR)]	24.5 (5.8)	25.4 (6.4)	25.5 (3.3)	
Waist circumference [cm, median (IR)]	90.5 (16)	91 (17.8)	95 (8.5)	0.687
CD4 cell count [cells/mm3, [median (IR)]	512.5 (336)	500 (311)	456 (509)	0.569
HIV RNA (<50) [*n* (%)]	138 (100)	44 (100)	10 (100)	
Hepatitis C co-infection [*n* (%)]	47 (30.9)	15 (28.8)	2 (16.7)	0.655
CDC clinical categories [*n* (%)]				0.252
A	90 (58.4)	24 (45.3)	5 (38.5)	
B	4 (2.6)	1 (1.9)	0 (0)	
C	60 (39)	28 (52.8)	8 (61.5)	
ART [*n* (%)]				
IP	77 (50)	36 (67.9)	10 (76.9)	0.023
NNRTI	82 (53.2)	17 (32.1)	3 (23.1)	0.005
NRTI	148 (96.1)	51 (96.2)	11 (84.6)	0.188
HOMA-IR index [median (IR)]	1.4 (1.5)	2.4 (2.5)	1.9 (3.2)	< 0.001
QUICKI index [median (IR)]	0.4 (0.1)	0.3 (0.1)	0.4 (0.1)	< 0.001
Total cholesterol [mg/dL, median (IR)]	224 (67)	224 (79)	234 (86)	0.743
LDL- cholesterol [mg/dL, median (IR)]	130 (70)	135 (66)	156 (79)	0.843
HDL- cholesterol [mg/dL, median (IR)]	49 (19)	44 (20)	52 (15)	0.314
Triglycerides [mg/dL, median (IR)]	186 (176.5)	223 (258.5)	170 (101)	0.131
Statin use [*n* (%)]	31 (20.1)	9 (17)	6 (46.2)	0.072
Fibrate use [*n* (%)]	43 (27.9)	21 (39.6)	1 (7.7)	0.061

*DM* diabetes mellitus, *OGTT* oral glucose tolerance test, *CL* clinical lipodystrophy, *cART* combination antiretroviral therapy, *BMI* body mass index, *ART* antiretroviral therapy, *PI* protease inhibitor, *NNRTI* non-nucleoside reverse transcriptase inhibitor, *NRTI* nucleoside reverse transcriptase inhibitor, *HOMA* homeostatic model assessment index, *QUICKI* quantitative insulin sensitivity check index, *SD* standard deviation, *IR* interquartile range

Patients diagnosed with DM were older than those with prediabetes, and these, in turn, were older than those without DM [55.00 (13.55) vs 50.36 (12.13) vs 43.38 (10.24) years; *p* < 0.001]. A progressive stage of glucose metabolism disorder appears related to the use of protease inhibitors [76.9% vs 67.9% vs 50%; *p* = 0.023], and the opposite is observed with the use of non-nucleoside reverse transcriptase inhibitors [23.1% vs 32.1% vs 53.2%; *p* = 0.005).

The HOMA-IR index was highest among the prediabetes group, and lowest among the no DM group [2.37 (2.49) vs 1.94(3.17) vs 1.41 (1.54); *p* < 0.001].

There were no differences between the diagnostic groups regarding sex, duration of HIV infection or ART use, presence or absence of clinical lipodystrophy, body composition types, BMI, waist circumference, CD4 cell count, percentage of viral suppression, prevalence of hepatitis C coinfection, CDC clinical category, lipid profile, and frequency of use of statins or fibrates.

### Fasting glucose

Forty four patients (20%) were diagnosed with prediabetes (100 mg/dL < fasting glucose< 126 mg/dL), and seven patients (3.2%) were diagnosed with DM (fasting glucose≥126 mg/dL). In Table 4, we present our results regarding the three categories of glucose homeostasis.

**Table 4:** Sample’s characteristics according to the presence of no DM, prediabetes and DM, accessed by fasting glucose

	Fasting glucose			*P* value
	<100 mg/dL	100–126 mg/dL	≥126 mg/dL	
*n* (%)	169 (76.8)	44 (20)	7 (3.2)	
Sex [*n* (%)]				0.049
Male	103 (60.9)	23 (52.3)	7 (100)	
Female	66 (39.1)	21 (47.7)	0 (0)	
Age [years, [median (IR)]	43 (15)	51.5 (15)	45 (14)	0.027
Duration of HIV infection [years, [median (IR)]	8 (6)	8 (5.8)	8 (6)	0.782
cART [years, [median (IR)]	6 (7)	6 (5.8)	8 (6)	0.408
Clinical lipodystrophy [*n* (%)]				0.082
Without CL	76 (45)	27 (61.4)	2 (28.6)	
With CL	93 (55)	17 (38.6)	5 (71.4)	
Body Composition [*n* (%)]				0.004
No lipodystrophy	26 (15.8)	4 (9.8)	1 (14.3)	
Isolated central fat accumulation	46 (27.9)	22 (53.7)	1 (14.3)	
Lipoatrophy	52 (31.5)	6 (14.6)	0 (0)	
Mixed form of lipodystrophy	41 (24.8)	9 (22)	5 (71.4)	
BMI [(kg/m2), [median (IR)]	24.4 (5.8)	26.1 (5.5)	26 (3.4)	0.052
Waist circumference [cm, [median (IR)]	88 (16.5)	95 (13.5)	95 (15)	0.005
CD4 cell count [cells/mm3, [median (IR)]	486 (344)	525 (298)	605 (382)	0.643
HIV RNA (<50) [*n* (%)]	151 (100)	35 (100)	6 (100)	
Hepatitis C co-infection [*n* (%)]	56 (33.5)	6 (14.3)	2 (28.6)	0.034
CDC clinical categories [*n* (%)]				0.398
A	89 (52.7)	27 (61.4)	3 (42.9)	
B	3 (1.8)	2 (4.5)	0 (0)	
C	77 (45.6)	15 (34.1)	4 (57.1)	
ART [*n* (%)]				
IP	96 (56.8)	23 (52.3)	4 (57.1)	0.885
NNRTI	79 (46.7)	20 (45.5)	3 (42.9)	0.999
NRTI	163 (96.4)	41 (93.2)	6 (85.7)	0.157
HOMA-IR index [median (IR)]	1.5 (1.3)	3.2 (2.6)	9.3 (6,8)	< 0.001
QUICKI index [median (IR)]	0.4 (0.1)	0.3 (0.04)	0.3 (0.04)	< 0.001
Total cholesterol [mg/dL, median (IR)]	220 (66)	240.5 (86)	234 (79)	0.061
LDL- cholesterol [mg/dL, median (IR)]	127 (67)	149 (62)	146 (74)	0.136
HDL- cholesterol [mg/dL, median (IR)]	47 (18)	49 (22)	41 (22)	0.651
Triglycerides [mg/dL, median (IR)]	189.5 (197.8)	214.5 (207.8)	184 (88)	0.975
Statin use [*n* (%)]	35 (20.7)	8 (18.2)	3 (42.9)	0.312
Fibrate use [*n* (%)]	50 (29.6)	15 (34.1)	0 (0)	0.200

*DM* diabetes mellitus, *CL* clinical lipodystrophy, *cART* combination antiretroviral therapy, *BMI* body mass index, *ART* antiretroviral therapy, *PI* protease inhibitor, *NNRTI* non-nucleoside reverse transcriptase inhibitor, *NRTI* nucleoside reverse transcriptase inhibitor, *HOMA* homeostatic model assessment index, *QUICKI* quantitative insulin sensitivity check index, *SD* standard deviation, *IR* interquartile range

We observed that there was a significant difference regarding sex between the diagnostic groups, as all the patients diagnosed with DM were men. In the prediabetes group, 47.7% of the patients were woman. The differences in age were also statistically significant, with the oldest patients being in the prediabetes group, and the youngest patients being in the no DM group [no DM 43.00 (15) vs prediabetes 51.50 (15) vs DM 45.00 (14) years; *p* = 0.027]. Regarding the different categories of body composition, we observe that patients without DM diagnosis had the highest proportions of patients in the categories “No lipodystrophy” (15.8%) and “Lipoatrophy” (31.5%), the prediabetes group had the highest percentage of patients in the “Isolated central fat accumulation” (53.7%) category, and the DM group had the highest percentage of patients in the category “Mixed form of lipodystrophy” (71.4%). Waist circumference was significantly lower in the no DM group [noDM 88.0 (16.5) vs prediabetes 95.0 (13.5) vs DM 95.0 (15.0); *p* = 0.005]. Patients with hepatitis C coinfection were more frequently classified as no DM group (33.5%), and less frequently in the prediabetes group (14.3%). The HOMA-IR index was highest among the DM group and lowest among the no DM group [no DM 1.45 (1.31) vs prediabetes 3.18 (2.57) vs DM 9.27 (6.79); *p* < 0.001].

There were no differences between the diagnostic groups regarding duration of HIV infection or ART use, presence or absence of clinical lipodystrophy, BMI, CD4 cell count, percentage of viral suppression, CDC clinical category, type of ART used, lipid profile, and frequency of use of statins or fibrates.

### Agreement analyses

Kappa coefficients were computed to estimate the agreement between the three diagnostic definitions. In Table 5 we present the results from the analyses between OGTT and HbA1c. The kappa coefficient value was 0.141 (*p* = 0.025), which corresponds to only a slight agreement. In Table 6, results from the agreement between fasting glucose and HbA1c are presented, and the kappa coefficient was 0.013 (*p* = 0.848). In Table 7, we present the results from the analyses between OGTT and fasting glucose. In this pair the kappa value was 0.206 (*p* < 0.001), which is considered to be a fair agreement.

**Table 5:** Analysis of the agreement between HbA1c and Glucose at 120 min during an OGTT

		Glucose at 120 min		Total
		<140 mg/dL	140–200 mg/dL	
HbA1c	<5.7%	140	42	182
	5.7–6.4%	14	11	25
Total		154	53	207
Kappa Coefficient = 0.141 (*p* = 0.025)				

*HbA1c* glycated haemoglobin, *OGTT* oral glucose tolerance test

**Table 6:** Analysis of the agreement between HbA1c and fasting glucose

		Fasting glucose		Total
		<100 mg/dL	100–126 mg/dL	
HbA1c	<5.7%	148	39	187
	5.7–6.4%	21	5	26
Total		169	44	213
Kappa Coefficient = 0.013 (*p* = 0.848)				

*HbA1c* glycated haemoglobin

**Table 7:** Analysis of the agreement between fasting glucose and Glucose at 120 min during a OGTT

		Glucose at 120 min			Total
		<140 mg/dL	140–200 mg/dL	≥200 mg/dL	
Fasting glucose	<100 mg/dL	128	34	7	169
	100–126 mg/dL	24	17	3	44
	≥126 mg/dL	2	2	3	7
Total		154	53	13	220
Kappa Coefficient = 0.206 (*p* < 0.001)					

*OGTT* oral glucose tolerance test

## Discussion

To our knowledge, this is the first study that has been carried out with HIV-infected patients, that combines the use of fasting glucose, OGTT and HbA1c, to establish the diagnosis of glucose homeostasis disturbances.

Analyzing the prevalence of DM and prediabetes throughout these different methods, we observe that the results varied considerably. Regarding the diagnosis of DM, no patients were identified when HbA1c was used, 13 patients (5.9% prevalence) when OGTT was used, and seven patients (3.2% prevalence) when fasting glucose was used. Prediabetes had a prevalence of 14.1% (31 patients) when using HbA1c, 24.1% (53 patients) when using OGTT, and 20% (44 patients) when using fasting glucose. Thus, we can conclude that HbA1c was the method that least diagnosed cases, and that OGTT was the one that diagnosed the most cases.

In accordance with our findings, HbA1c has been found to underestimate glycemic levels in HIV-infected patients when compared with other types of diagnostic methods, in several studies [4, 17–22]. Possible explanations for the lower than expected HbA1c values in these patients have been hypothesized. For example, low hemoglobin values [19], and situations that shorten erythrocyte lifespan, such as hemolysis or some hemoglobinopathies, have been associated with lower HbA1c values [18]. Diop et al. have found that the discordance HbA1c-fasting glucose was positively correlated with the mean cell volume, and that hemolysis, diagnosed by a very low haptoglobin level, had a higher prevalence in the HIV-infected patients [18]. In fact, this relationship between mean cell volume and HbA1c-fasting glucose discordance has been observed by several authors [17, 18, 21–23], and Glesby et al. validated it when they observed that higher mean cell volume values emerged as the single most important factor associated with a lower HbA1c than predicted by fasting glucose. High mean cell volume, as a marker of a greater proportion of younger erythrocytes that had a shorter time to become glycated, suggest a greater red blood cell turnover in the HIV-infected patients [17]. The eventual relationship with drugs used in the treatment of HIV infection with these hematologic findings is difficult to study, as ART is generally used in combination.

OGTT was the method that diagnosed the highest percentage of patients with DM and prediabetes. Similar results have been found in the literature [24–26]. Gianotti et al. demonstrated in their study that OGTT revealed that 11% of their cohort with long-standing HIV infection had prediabetes or DM, undiagnosed on the basis of fasting glucose levels alone [24], while Seang et al. detected a 31% relative increase in the prevalence of DM diagnosis among HIV-infected women [25]. Epidemiological evidence has also supported this observation in the general population, especially among older patients [27]. In light of this knowledge, the 2017 European AIDS Clinical Society Guidelines recommend that HIV-infected patients with a fasting glucose diagnosis of prediabetes should carry out an OGTT in order to identify overt diabetes [10].

With regards to the characteristics that differed significantly between the diagnostic groups, we observed that there were some that were important across all three methods, while others varied specifically, depending on the method used for the assessment.

Sex was a factor that showed association with the diagnosis only when fasting glucose was used. With this method, the total number of patients that had the diagnosis of DM were male, and there was also a higher prevalence of male (52.3% versus 47.7% female) in the prediabetes group. Some authors have reported a lack of significant differences between sexes when considering the risk factors for glucose homeostasis disturbances in HIV patients [24], while others, in agreement with our findings, stated that male sex was associated with increased risk of new-onset DM [5, 28].

In all three methods, a progressive stage of glucose homeostasis disturbance was associated with older age. This finding is congruent with the great majority studies done on the subject [1–5, 7, 28, 29]. This has been highlighted in recent literature that, after the introduction of ART (which has dramatically reduced HIV-related mortality and morbidity, substantially increasing longevity), HIV-infected individuals have a potential of developing metabolic complications, which is comparable to that of the general population [1]. In these patients, the importance of traditional cardio-metabolic risk factors should be emphasized, as these are likely to exert an equal influence on HIV-infected patients as they do in the general population [1]. On the other hand, there have been studies that suggest that the aging process might be premature or accelerated in these patients, leading to the manifestation of metabolic complications earlier in life [2]. This highlights the importance of closely monitoring for the development of cardio-metabolic abnormalities in these patients.

Regarding the duration of HIV infection and ART use, we observed that, regardless of the diagnostic method used, no significant difference between the groups was observed. This contrasts with findings in the literature, that suggest that a higher prevalence of DM is associated with a higher duration of HIV infection and ART use [1, 3, 30], but, is in agreement with a study done by Araújo et al., in which also, no association between the duration of infection and the development of glucose homeostasis disturbances was found [31].

BMI was considered to be significantly higher in prediabetes patients, compared to the no DM patients, but only when HbA1c was used for the assessment. This finding is corroborated by several authors [3–5, 7, 28–30] who described a significant association between a higher BMI and the presence of disturbances of glucose homeostasis in HIV-infected patients. Additional to the BMI, abdominal fat accumulation or trunk obesity has been identified as a factor primarily associated with the prevalence of disturbances of glucose homeostasis in HIV-infected patients [8, 25, 26, 30–33], just as described in the general population. In our study, when fasting glucose was used for the assessment of diabetes, there was a significant association between waist circumference and the diagnosis. This parameter was higher in DM and prediabetes patients, when compared with the no DM ones. Furthermore, in this diagnostic method, we obtained significant differences regarding the classes of body composition, with most significant differences observed in the isolated central fat accumulation (highest percentage of patients in the prediabetes group) and mixed forms of lipodystrophy (highest percentage of patients in the DM group), which mirror the effect of these higher waist circumference levels in these diagnostic groups. In fact, it has been reported that abdominal fat accumulation is a major contributor to glucose metabolism disturbances when compared to lipoatrophy associated with the acquired lipodystrophy of HIV infection [8]. Endocrine activity of adipose tissue takes a central place in the pathogenesis of metabolic disorders [34], thus it makes sense, pathogenically, that this relation can be observe. There were no differences in the diagnostic groups regarding the presence of absence of clinical lipodystrophy, in neither of the diagnostic methods.

Hepatitis C coinfection has been found to be associated with the development of glucose homeostasis disturbances among HIV-infected patients [7, 29, 31]. In our study, when fasting glucose was used for assessment, Hepatitis C coinfection had a prevalence of 33.5% (56 patients) in the no DM group, 14.3% (6 patients) in the prediabetes group, and 28.6% (2 patients) in the DM group. Non-diabetic patients had the highest percentage of coinfection. However, we have to keep in mind that in the prediabetes and DM group, the number of patients is very reduced. Hence, the small sample size may be a limitation for the interpretation of this finding.

The association between ART and the development of diabetes has been frequently described throughout the literature [1, 4, 5, 26]. When we analyzed the type of ART used, only when the OGTT method was applied, were there significant differences. In this group, we observed that progressive stage of glucose homeostasis disturbance was positively associated with the use of protease inhibitors, and negatively associated with the use of non-nucleoside reverse transcriptase inhibitors. It has in fact been reported that protease inhibitors-based regimens might be associated with the development, and/or acceleration of the progression of metabolic complications [1, 30], although this association is not consensual among the literature [5, 7, 31, 33, 35, 36]. Slama et al. reported that certain ARTs (protease inhibitors, non-nucleoside reverse transcriptase inhibitors and zidovudine) were associated with the HbA1c-fasting glucose discordance [22], however, specifically regarding the use of HbA1c for the assessment, similar to our findings, Kim et al. reported a lack of association between all types of ART and the diagnosis of diabetes [37]. In other studies, non-nucleoside reverse transcriptase inhibitors were recognized as being an alternative regimen to protease inhibitors in patients with metabolic complication, as these had a lower prevalence of such complications [1, 36]. On the other hand, nucleoside reverse transcriptase inhibitors have been associated with an increased risk of glucose metabolism disturbances [5, 28, 31, 35, 36], and with HbA1-fasting glucose discordance in the assessment of HIV-infected patients, suggesting that, in these patients, HbA1c should not be used for the assessment of glycaemia [21]. In our study, there was no significant association between the use of nucleoside reverse transcriptase inhibitors and the development of glucose homeostasis disturbances. It is, however, extremely difficult to determine which drug is responsible for the increased risk of glucose metabolism disturbances, as patients usually use these pharmacological solutions in combination with each other, and, furthermore, therapeutic changes often occur during the course of the disease.

Despite the euglycemic insulin clamp technique being the gold standard technique for the study of tissue sensitivity to insulin [38], we used easier-to-perform methods - HOMA-IR and QUICKI indexes, that have shown a strong correlation with the gold standard, and good correlation between each other, which allowed us to have a robust estimate of insulin sensitivity [15, 16, 24]. The HOMA-IR index median levels were higher in the prediabetes group, when compared to the no DM group when we used the HbA1c, lowest in the no DM group and highest in the DM group when we used fasting glucose, and highest in the prediabetes group and lowest in the no DM group when we used OGTT. These results support the evidence that progressive stages of glucose metabolism disorders are associated with progressive stages of insulin resistance. In the case of OGTT, where the prediabetes group had indexes that were more altered than the DM group, a possible explanation may be that, in a DM state, insulin deficiency is superior to that of the prediabetes state, or this may be due to a small sample in the DM group. Hence, in both prediabetes and DM, we have insulin resistance, however, as insulin deficiency is greater in the DM state, the calculation of these indexes is affected, becoming less pronounced.

There were no differences in HIV-related parameters between the different groups of glucose homeostasis disturbances. Research has associated a lower CD4 cell count with higher prevalence of glucose homeostasis disturbances [3, 24], and a count of <500 cells/mm^3^ was found to be strongly associated with HbA1c-fasting glucose discordance [22]. Although we had, throughout our study, groups that registered CD4 cell counts below this level, these were never statistically significant. Thus, we can hypothesize that this characteristic had a small impact on the development of this kind of complication.

In our agreement analyses, we used the kappa coefficient, which is a robust statistic, useful for either interrater or intrarater reliability testing. Kappa values are considered to represent a slight agreement when they are between 0 and 0.20 and fair agreement when they are between 0.21 and 0.40 [39]. Hence, in face of our results, we can conclude that the agreement between fasting glucose-OGTT was fair (0.206), and between fasting glucose-HbA1c (0.013) and OGTT-HbA1c (0.141) was slight. Consequently, we can say that these diagnostic methods agree much less than would be expect only by chance.

However, the ability to use HbA1c to screen HIV-infected patients in a nonfasting state and for estimating long-term glycaemia makes it a very useful tool for the management of DM [23]. Several authors have shown that HbA1c underestimates glycaemia levels in these patients, making it a less accurate diagnostic method [4, 17, 18, 21, 22]. In fact, Eckhardt et al. have shown HbA1c to be very insensitive, but highly specific for the diagnosis of DM in HIV patients [23]. In light of this evidence, the 2017 American Diabetes Association guidelines state that this test underestimates glycaemia in HIV-infected individuals, and that it is not recommended for diagnosis, and even for monitoring, which presents challenges [40]. On the other hand, OGTT has been shown to reveal cases of prediabetes, and even DM, in individuals with normal fasting glucose levels [24, 25]. Thus, the use of this test might be an effective method for detecting these disturbances of glucose metabolism prematurely, making it possible to avoid the severe complications of the disease by means of an early diagnosis [24].

Due to the fact that the HIV population has the potential to develop cardio-metabolic abnormalities through multiple pathways, determining the magnitude of DM in this population highlights the need for preventive and management strategies. Overall, the optimal diagnostic algorithm is still poorly defined, and the question remains as to whether clinicians should use direct measures of glycaemia, or HbA1c to achieve a more accurate diagnosis.

### Limitations

This study had some limitations, mainly related to the cross-sectional nature of our analyses, which prevents us from making any conclusions regarding causality. Although we included all patients referred to our department, we cannot exclude bias in the referral, as some patients could have been referred because there was already some suspicion of them having DM or other metabolic disorder. Therefore, we might have selected a study population with a different distribution of metabolic and endocrine complications, compared to the general patients’ population. Consequently, these results cannot be extrapolated for the total HIV population.

## Conclusions

In our study, it was clear that disturbances of glucose homeostasis was a prevalent problem, specifically prediabetes, which had values of 14.1, 24.1, and 20% prevalence, depending on the method used for diagnosis. Hence, more sensitive diagnostic tools are essential for prevention of DM complications in this population. Our results indicate that HbA1c underestimated glycaemia levels, and that OGTT might, in fact, allow for an earlier diagnosis of glucose homeostasis disturbances, as this was the method that had a higher capacity to detect them. Therefore, in the HIV population, it would be prudent for medical practitioners to use direct measures of glycaemia (fasting glucose or OGTT) to diagnose glucose homeostasis disturbances, or, perhaps, even consider establishing lower HbA1c thresholds to determine this diagnosis.

## Abbreviations

ADA: American Diabetes Association
AIDS: Acquired immune deficiency syndrome
ART: Antiretroviral therapy
BMI: Body mass index
CDC: Centers for disease control and prevention
DM: Diabetes mellitus
HbA1c: Hemoglobin A1c/glycated Hemoglobin
HDL: High density lipoprotein
HIV: Human immunodeficiency virus
HOMA: Homeostatic model assessment
LDL: Low density lipoprotein
OGTT: Oral glucose tolerance test
QUICKI: Quantitative insulin sensitivity check index
